# Ribonucleoproteins in Archaeal Pre-rRNA Processing and Modification

**DOI:** 10.1155/2013/614735

**Published:** 2013-03-10

**Authors:** W. S. Vincent Yip, Nicholas G. Vincent, Susan J. Baserga

**Affiliations:** ^1^Department of Molecular Biophysics and Biochemistry, Yale University School of Medicine, New Haven, CT 06510, USA; ^2^Department of Microbial Pathogenesis, Yale University School of Medicine, New Haven, CT 06510, USA; ^3^Department of Therapeutic Radiology, Yale University School of Medicine, New Haven, CT 06510, USA; ^4^Department of Genetics, Yale University School of Medicine, New Haven, CT 06510, USA

## Abstract

Given that ribosomes are one of the most important cellular macromolecular machines, it is not surprising that there is intensive research in ribosome biogenesis. Ribosome biogenesis is a complex process. The maturation of ribosomal RNAs (rRNAs) requires not only the precise cleaving and folding of the pre-rRNA but also extensive nucleotide modifications. At the heart of the processing and modifications of pre-rRNAs in Archaea and Eukarya are ribonucleoprotein (RNP) machines. They are called small RNPs (sRNPs), in Archaea, and small nucleolar RNPs (snoRNPs), in Eukarya. Studies on ribosome biogenesis originally focused on eukaryotic systems. However, recent studies on archaeal sRNPs have provided important insights into the functions of these RNPs. This paper will introduce archaeal rRNA gene organization and pre-rRNA processing, with a particular focus on the discovery of the archaeal sRNP components, their functions in nucleotide modification, and their structures.

## 1. Introduction

Without ribosomes, cells cannot grow and divide. Thus, it is not surprising that ribosomes have been selectively conserved through millennia and are one of the few macromolecular machines present in all three domains of life. Having been under such tight selective pressure, ribosomes have been and continue to be used in establishing phylogenetic relationships through one specific component, the 16S rRNA in Bactria and Archaea and the 18S rRNA in Eukarya [1–3]. This led scientists to uncover that instead of only “prokaryotes” and “eukaryotes,” there is an additional third domain that would become the Archaea [4, 5].

Ribosome biogenesis and processing begins with ribosomal gene organization. This paper will cover both the genome organization of various rRNA operons in Archaea as well as the small ribonucleoproteins (sRNPs) responsible for chemical modification of nucleotides on the rRNA. In particular, the two most abundant types of chemical modifications are mediated by box C/D and box H/ACA sRNPs. It is not yet well understood why these nucleotides are chemically modified. In considering these modifications of rRNA, it is first important to understand why ribosomes are essential, how Archaea are unique, and how their ribosomal RNA loci are organized in the genome. 

## 2. Ribosomes and rRNA

Ribosomes are essential for the translation of mRNA into a polypeptide chain that is then folded into a functional protein [6]. These cellular machines are not only comprised of rRNA (transcribed from ribosomal DNA, rDNA), but also a number of ribosomal proteins that are required for the correct structure and function of the ribosome [7]. Ribosome biogenesis is a very energy-intensive process [8] and requires a host of proteins and RNA modifications. In an actively growing *Saccharomyces cerevisiae* cell, transcription of the rRNA genes can account for up to 60% of total cellular transcription; additionally, the processing of ribosomal proteins takes up to 90% of mRNA splicing activities within the actively growing cell [8]. Considering that ribosomes are essential, it is not surprising that they are selectively conserved. As previously mentioned, rRNA has proven to be very helpful in establishing evolutionary relationships of almost all known organisms, both microscopic and macroscopic [1–3].

## 3. Ribosomal Architecture

All ribosomes are comprised of a large and small subunit; while the subunits differ in structure across the evolutionary domains, they share a common function. The small subunit (SSU) contains one rRNA in all organisms (16S in Bacteria and Archaea, 18S in Eukarya). Each also has a large subunit (LSU) that in eukaryotes is comprised of the 28S (25S in *S. cerevisiae*), 5.8S, and 5S rRNAs, while in Bacteria and Archaea is composed of the 23S and 5S rRNAs (Table 1). In addition to these rRNAs, ribosomal proteins account for part of the mass of each subunit. For example, the prokaryotic SSU has a 16S rRNA, but sediments at 30S in a sucrose gradient. While the 70S prokaryotic ribosome contains a 50S LSU and 30S SSU, the eukaryotic ribosome is 80S, with a 60S LSU and 40S SSU [10].

## 4. Archaea: An Independent Monophyletic Grouping

**Figure 1:**
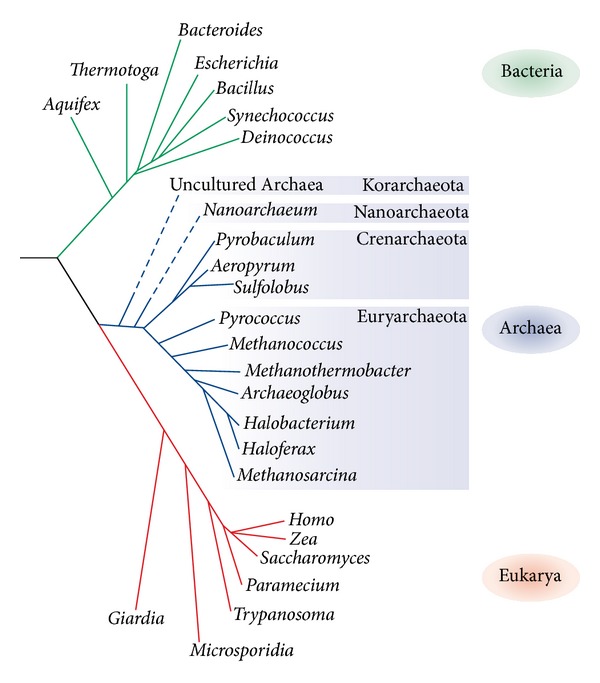
Phylogenetic relatedness of the three domains of life and of major phyla. Reprinted with permission from [9]. The Korarchaeota and Nanoarchaeota, as putative phyla, are shown via dotted lines. The Thaumarchaeota are not included, as they were previously grouped with the Crenarchaeota, and their independent phylogeny has not been unanimously agreed upon.

Relative to rRNA sequence, Archaea are phylogenetically distinct from Bacteria and Eukarya (Figure 1). Archaea have ribosomes that are structurally similar to bacterial ribosomes: the LSU contains a 23S and 5S rRNA while the SSU contains a 16S rRNA. While the domains Bacteria and Eukarya are well studied and understood, the members of Archaea remain more of a mystery, especially considering that organisms with extreme growth requirements are often contained within the domain. Hyperthermophilic, psychrophilic, halophilic, and even piezophilic organisms are all contained within the Archaea.

Considering the diversity of organisms that comprise the archaeal domain, it is not surprising that they represent strong candidates for biological research. It is essential that scientists continue to emphasize that the Archaea do in fact stand alone as a domain and contain phyla and biological divisions that are just as structured and inclusive as the other domains (Figure 1, Table 1). With biochemical and structural features that are so unique to Archaea (i.e., cell wall and lipid differences between Archaea and the other domains; see [12] for an early account of the differences) it is easy to understand that they represent a wealth of knowledge waiting to be uncovered. One particular area of interest lies in biotechnological applications of Archaea [13].

## 5. rRNA Operon Number and Organization across Archaea 

### 5.1. rRNA Operon Number across Archaea

Within the Archaea, there is great variation in the number of *rrn *genes encoding the 23S, 16S, and 5S ribosomal RNAs. There are one to four copies of the 16S *rrn* gene in diverse archaeal species, with over half of the sequenced genomes having only one, which lies in contrast to the one to fifteen copies that can be found in bacterial species [14, 15]. Specifically, approximately 20% of sequenced bacterial genomes have one copy of the 16S *rrn* gene, while 11% have greater than eight [14, 15]. The fact that there is often more than one repeat of these genes in organisms that typically are monoallelic for other genes underlies the importance of the *rrn* genes and the essential requirement for an abundant supply of properly functioning ribosomes. Additionally, all sequenced Crenarchaeota, Nanoarchaeota, and Korarchaeota have only one copy of the 16S rRNA gene, so that any species with more than one copy of the 16S rRNA gene belongs to the Euryarchaeota [14]. Although rDNA gene number and organization varies across archaeal species, the reason for such organization remains unknown.

### 5.2. Polycistronic Transcription of rRNA Genes in Archaea

**Figure 2:**
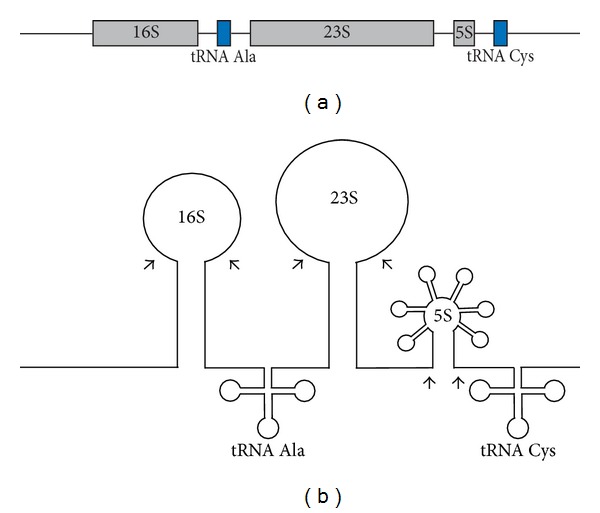
An example of a polycistronic rDNA locus in Archaea. (a) Linear representation of the genome organization of the *rrn* locus typical of the Euryarchaeotes. There are also various other combinations of operon organization (see text). (b) Secondary structure of pre-rRNA transcript in the Euryarchaeotes. Splicing occurs at arrow points to liberate the individual constituents from the polycistronic transcripts via an archaeal-specific mechanism. Only splice sites for individual rRNA components are included for simplicity. Adapted with permission from [11].

In several species of Archaea, the 16S, 23S, and 5S rRNAs exist in a continuous polycistronic operon. In this organization, the 16S, 23S, and 5S rRNA genes are transcribed as a single transcript and then excised via an archaeal-specific splicing mechanism (Figure 2). An example of the polycistronic transcription of rRNA operons is in the model archaeon, *Haloferax volcanii. *Intriguingly, *H. volcanii* has two full copies of the rRNA operons that each contains a copy of the 16S, 23S, and 5S rRNAs [18]. Another example of polycistronic transcription of rRNA genes is in *Halobacterium cutirubrum*, which has been reported to contain a single copy of rRNA genes encoded in one operon encoding the 16S, tRNA^Ala^, 23S, 5S, tRNA^Cys^ [19]. This setup is typical of what is seen in the Euryarchaeota (Figure 2) [11]. As in *H. cutirubrum*, the archaeal rRNA operons often contain tRNA genes interspersed between the rRNA genes. This represents a clear way to ensure that certain tRNA genes are transcribed. For information on the presence, number, and organization of tRNA genes, see [14, 15]. 

### 5.3. Partial Uncoupling of Transcription of rRNA Genes in Archaea

While a polycistronic operon would seem to be an efficient way to transcribe archaeal rRNAs, other gene organizations exist as well. There is no shortage of examples that represent variations on a theme of rRNA gene organization in Archaea. For example, *Thermoproteus tenax* and *Desulfurococcus mobilis*, both members of the Crenarchaeota, have an operon containing the 16S, and 23S rRNA genes but not including the 5S genes, which are found under the control of a different promoter at an alternative locus in the genome [44, 45]. *Sulfolobus solfataricus,* a model archaeal organism, also has this operon architecture [46]. Moreover, *Thermus thermophilus* processes the 23S and 5S rRNA genes together within an operon, with the 16S rRNA gene located elsewhere in the genome [47]. Most interesting of the partially uncoupled operons is *Methanococcus jannaschii, *which contains two copies of the genes for the 16S, 23S, and 5S rRNAs. In this species, there is one 16S/23S/5S operon (Operon A) and a 16S/23S operon (Operon B) that is uncoupled from the second 5S gene [48]. 

### 5.4. Complete Uncoupling of Transcription of rRNA Genes in Archaea

Some archaeal species do not have any operon organization of their rRNA genes but instead have three separate genes at three separate locations under three separate promoters, each encoding the 23S, 16S, and 5S rRNAs. Examples of such species are *Thermoplasma acidophilum*, the first archaeal organism to be discovered with this rRNA genome organization [49], and *Nanoarchaeum equitans* [50]. It should be noted that in *N. equitans*, there is also a dispersal of ribosomal proteins throughout the genome that are found in close proximity not only in other archaeal species of other phyla, but also in the Bacteria [51]. In *N. equitans*, the uniqueness of this organism as the only known member of the Nanoarchaeota phylum can explain the unique genome organization, but this alone cannot be used as an umbrella explanation for the separate 23S, 16S, and 5S organization since it is also found in *T. acidophilum*. 

Since all of the rRNA components (i.e., 23S, 16S, and 5S) are required to assemble a functioning ribosome, what are the reasons, if any, in uncoupling transcription in each component in the rRNA unit? Similarly, what are the reasons for partially uncoupling the operons as is seen in *M. janaschii*? Further research will be required to understand archaeal rRNA gene organization and why some organisms have polycistronic transcription, while others have transcriptional uncoupling of rRNA synthesis.

## 6. Introns within Archaeal rRNA Genes

In addition to variation in the number and organization of the rRNA genes throughout genomes, there is also variation amongst Archaea in terms of intronic sequences found within the rRNA sequences. While Bacteria do not contain introns within the genes for the 23S, 16S, and 5S RNAs, some archaeal species, in fact, do. Intronic sequences in the 23S ribosomal RNA gene were first described in *Desulfurococcus mobilis* with a mechanism of splicing unique to Archaea [20]. Interestingly, there have even been reports of 23S rRNA genes containing two introns of approximately equal length in *Staphylothermus marinus* [21]. Moreover, there have also been reports of introns found within the 16S rRNA gene. The presence of an intron in *Pyrobaculum aerophilum*, but not the closely related *Pyrobaculum islandicum,* was the first report of an intron found in a 16S rRNA gene [22].

Many of these archaeal rDNA introns contain putative open reading frames that often lack sequence similarity to known proteins [20–22]. There have, however, been reports of these sequences coding for proteins, such as the site-specific endonuclease I-DmoI found in the intron of the 23s rRNA of *Desulfurococcus mobilis *[23, 24]. More research will be required to see whether there are additional examples of functional proteins or noncoding RNAs produced from these introns or whether these are simply few isolated cases. Their origin is also interesting considering that Bacteria do not contain intronic sequences in these genes.

Excision of introns in Archaea takes part through an archaeal-specific splicing mechanism [53] via-archaeal specific endonucleases, which is unusual since Archaea do not contain Group I or Group II introns [54]. The hallmark of this splicing reaction is the recognition of the bulge-helix-bulge motif that is found at the intron-exon junction [38, 53] (for more on archaeal splicing, see [11, 21, 53–55]). The presence of introns in archaeal rRNA genes suggests that, while the ribosomes and rRNAs are more similar to the Bacteria in structure, their processing and even, at times, gene organization (i.e., presence of introns in the rRNA genes) is more similar to Eukarya. This is one of many aspects of Archaea that truly identifies the domain as independent and part of neither the bacterial nor eukaryotic groupings. 

## 7. RNA-Guided rRNA Modifications and Guide Small RNPs in Archaea

Certain rRNA processing events in Archaea are similar to those in Eukarya. Indeed, similar to eukaryotic rRNAs, archaeal rRNAs are heavily modified. rRNAs from eukaryotes such as *S. cerevisiae* contain ~100 modifications (Table 1) [34, 37–40]. Archaeal rRNAs contain a comparable number of modifications [36–38, 56]. For instance, *S. solfataricus* has ~70 modifications in the rRNAs [36]. In direct contrast, bacterial rRNAs are modified to a much lesser extent, containing merely ~40 modifications (most of which are base modifications) [34, 37, 38, 40, 41].

**Figure 3:**
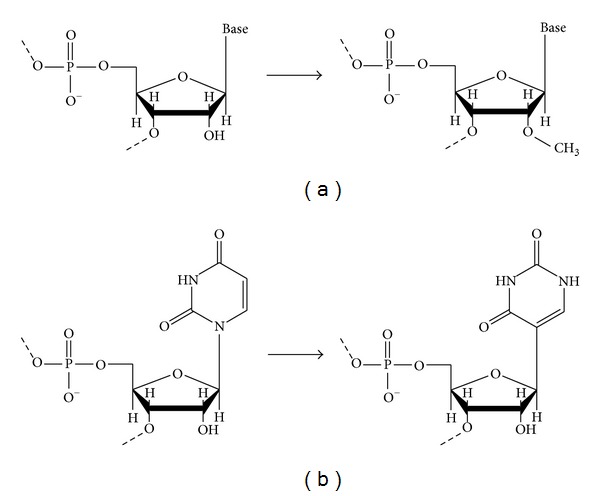
Two major chemical modifications in archaeal rRNAs. (a) In 2′-*O*-methylation, a methyl group is added to the 2′ hydroxyl of the nucleotide ribose. (b) In pseudouridylation, the uridine base is isomerized.

The two major types of chemical modifications in rRNAs are the methylation of the 2′ hydroxyl group, known as 2′-*O*-methylations (Nms), and the isomerization of the uridine base into pseudouridine, known as pseudouridylation (Ψ) (Figure 3). There are two mechanisms for chemically modifying rRNA: site-specific enzyme catalysis or RNA-guided catalysis [29–35]. While all of the rRNA chemical modifications in Bacteria are catalyzed by site-specific enzymes, most modifications in Archaea are achieved by RNA-guided RNP complexes [34, 35, 56–59]. 

In fact, these RNA-guided nucleotide modification machines are only present in the Archaea and Eukarya and absent in Bacteria [26–35, 56–60]. These machines, known as small ribonucleoproteins (sRNPs) in Archaea and small nucleolar ribonucleoproteins (snoRNPs) in Eukarya, are named after the small RNAs (sRNAs) in Archaea and small nucleolar RNAs (snoRNAs) in Eukarya. There are two types of s(no)RNPs: box C/D s(no)RNPs and box H/ACA s(no)RNPs, which carry out 2′-*O*-methylation and pseudouridylation, respectively [26–28, 30–33, 60]. Within a functional type of sRNP, the protein components are the same but the sRNAs differ. Thus, the sRNA provides the specificity of the nucleotide modification [26–28, 30–33, 60]. Hence, the sRNAs are often referred to as guide RNAs. 

## 8. Discovery of Archaeal sno-Like RNAs

The discovery that archaeal rRNAs contain a comparable number of chemical modifications as eukaryotic rRNAs prompted a search for sno-like guide RNAs (sRNAs) in Archaea. Both computational and experimental approaches have been used to detect guide sRNAs in various archaeal species [42, 43, 61–67]. Computational approaches used to identify these sRNAs take advantage of the conserved length and sequence elements and homologies with known snoRNAs or sRNAs in other eukaryotes and Archaea [63, 65, 67]. However, the relatively small sizes of the conserved sequence features of the sRNAs and the low degree of conservation of the sequence outside these features have presented challenges to the identification of sRNAs in Archaea. Therefore, other algorithms have been developed to identify candidate sRNAs [62, 67].

 Alternatively, experimental approaches can be used to search for archaeal sRNAs. Some approaches make use of Sanger sequencing of a size-selected cDNA library derived from total archaeal RNA [42, 64]. Others take advantage of the fact that the guide sRNAs form sRNP complexes and use immunoprecipitation methods to isolate the sRNAs [43, 61]. Recently, advances in nucleic acid sequencing technologies have allowed the direct sequencing of the selectively enriched stable RNA species from total RNA of *N. equitans* [66]. 

The combination of computational and experimental approaches presents a powerful means to identify putative guide sRNAs. These approaches have led to the discovery of over one hundred candidate guide sRNAs from various archaeal species in the Euryarchaeota, Crenarchaeota, and Nanoarchaeota phyla [42, 43, 61–67]. Interestingly, in approaches where both box C/D and H/ACA sRNAs were identified, the number of sRNAs of the H/ACA type is usually fewer than those of the C/D type [43, 64, 66]. This correlates with the finding that archaeal rRNAs usually have fewer pseudouridylations than 2′-*O-*methylations [35, 36, 38]. Alternatively, however, these may also be a result of the bias inherent in the approaches being used [67]. 

Whether the majority of archaeal guide sRNAs can assemble into catalytically active sRNPs is not known. The existence of many candidate sRNAs discovered using experimental approaches has been confirmed by either northern blots or by primer extension [42, 43, 61–65]. Some predicted sRNAs have been shown experimentally to be able to assemble into sRNPs which are catalytically active [26, 43, 65, 67–69]. In addition, the predicted target sites of some candidate guide sRNAs have been confirmed to be modified [42, 43, 61, 63–65, 67]. In contrast, many putative sRNAs are orphan sRNAs with no known predicted target sites [42, 43, 61–67], so it remains unclear whether they are *bona fide* guide sRNAs. Moreover, because current computational and experimental approaches rely heavily on previously known structures and sequence features of the sRNAs, many potential guide sRNAs may have been overlooked [67]. Nevertheless, the identification of candidate sRNAs permits a general understanding of their sequence and structural features and is the first step in characterizing them and their sRNPs in Archaea. 

## 9. Properties and Secondary Structures of Box C/D sRNA

**Figure 4:**
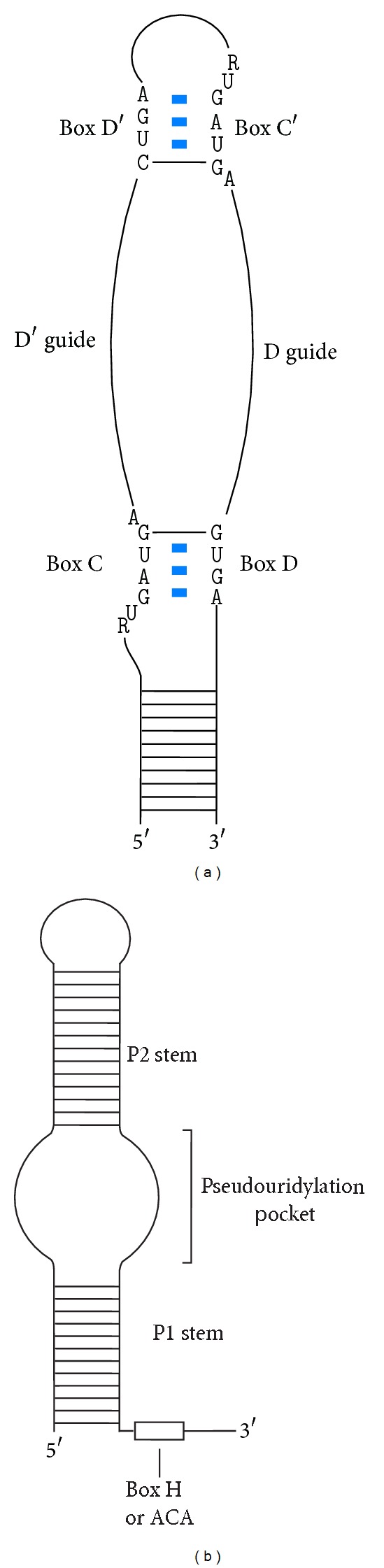
Secondary structures of archaeal guide sRNAs. (a) The secondary structure of box C/D sRNA. The box C/D and the box C′/D′ motifs form the kink-turn and kink-loop motif, respectively. The two unpaired strands of sequences flanked by the box C/D and box C′/D′ motifs base-pair with substrate and are called guide sequences. (b) The secondary structure of a single-hairpin box H/ACA sRNA. The 3′ end of the stem is immediately followed by a box H or box ACA motif. The pseuduouridylation pocket is in the middle of the hairpin with two unpaired strands of sequences that are capable of substrate base-pairing.

Archaeal box C/D sRNAs are typically ~50–60 nt long [42, 43, 59, 61, 64, 66]. The sRNA is a critical component in the catalysis of 2′-*O*-methylation. It contains two consensus sequence elements, boxes C (RUGAUGA) and D (CUGA), located near the 5′ and 3′ termini of the RNA, respectively (Figure 4(a)) [42, 43, 56–59, 61–64, 66]. In addition, it also contains two similar sequence elements, C′ and D′, in its internal region [42, 43, 56–59, 61–64, 66]. Boxes C and D form noncanonical GA:AG sheared pairs [70]. The 5′ region of box C is a 3 nt asymmetric bulge. The GA:AG sheared pairs and the 3 nt asymmetric bulge allow the formation of a kink between the noncanonical stem formed by the box C/D motif and the canonical stem formed between the 5′ and 3′ termini [70, 71]. This motif is known as the kink-turn motif and is a structural feature of the box C/D sRNA [70, 71]. Similarly, boxes C′ and D′ also form a kink-turn-like motif [72]. However, instead of a stem-kink-stem structure, the boxes C′/D′ form a stem-kink-loop structure, known as the kink-loop motif [72]. 

The C/D and C′/D′ motifs are separated by two unpaired strands of RNA [42, 43, 61–67]. These regions contain the guide sequences. The guide sequences 5′ to box D and to box D′ are, respectively, known as the D and the D′ guides. They form Watson-Crick base-pairs with the substrate RNAs [26, 31–33, 37, 56, 58, 59]. Base-pairing between the guide RNA and its substrate(s) allows the substrate(s) to be specifically 2′-*O*-methylated at a site that is five nucleotides 5′ from box D or D′. Intriguingly, in the several archaeal species examined, the unpaired sequences separating the C/D and C′/D′ motifs have a conserved length of 10–12 nt [73]. The maintenance of this length has been demonstrated to be essential for 2′-*O*-methylation [73].

## 10. Box C/D sRNPs

**Figure 5:**
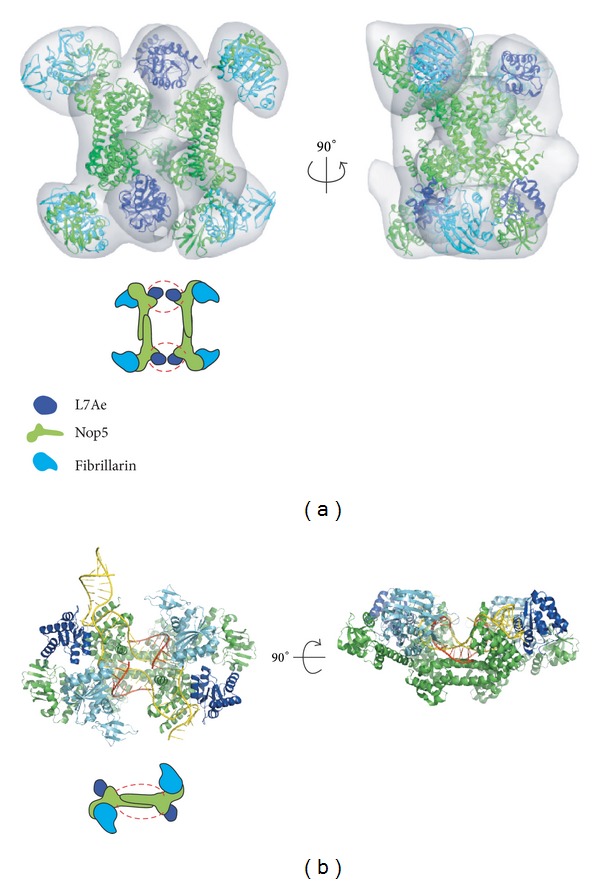
Structures of archaeal box C/D sRNPs. (a) Top panel, two views of negatively stained EM reconstruction of *S. solfataricus* box C/D sRNP reconstituted with a natural sRNA (adapted from [16]). Crystal structures of the core proteins, L7Ae (blue), Nop5 (green), and fibrillarin (cyan), are docked into the volume. Bottom panel, schematic of the di-sRNP model. (b) Top panel, two views of the X-ray crystal structure of *S. solfataricus* box C/D sRNP reconstituted with a nonnatural sRNA, rendered in PyMol (PDB ID: 3PLA [17]). Core proteins are color-coded as in (a). sRNA is in yellow and the substrate RNA is in red. Bottom panel, schematic of the mono-sRNP model.

 Box C/D s(no)RNPs carry out site-specific 2′-*O*-methylation of pre-rRNAs. The minimal components required to make a functional box C/D s(no)RNP were revealed when a catalytically active box C/D sRNP was reconstituted *in vitro* for the first time using recombinant *S. solfataricus* proteins and *in vitro* transcribed *S. acidocaldarius* sRNA by Omer et al. [26]. The catalytically active sRNP is composed of three protein components, L7Ae, Nop5, and fibrillarin, and an sRNA component (Figure 5) [26]. These three proteins and the sRNA constitute the core sRNP complex that is the minimal set of components necessary for catalytic activity *in vitro *[26]. It is unclear whether other factors associate with the core complex for optimal assembly or catalytic activity *in vivo *[26]. 

Since the first *in vitro *reconstitution of the core box C/D sRNP, its structure and biochemistry have been extensively studied [16, 17, 26, 68, 70, 73–90]. The L7Ae protein, which is also a large ribosomal subunit protein in Archaea, binds the kink-turn or kink-loop motif of the sRNA formed by boxes C/D and by boxes C′/D′ [17, 26, 70, 75, 76, 78, 79, 86, 87]. Each individual kink-turn or kink-loop motif allows the binding of one L7Ae protein [78, 79]. Therefore, one single sRNA allows the binding of two copies of the protein. The binding of L7Ae is required for the assembly of Nop5, and fibrillarin [26, 78, 79]. Without the binding of L7Ae, Nop5 and fibrillarin have little to no binding affinity for the sRNA [26, 79]. Nop5 contains a coiled coil domain that allows homodimerization [77, 81, 84]. It also interacts with a fibrillarin *in vitro* to form a Nop5/fibrillarin heterodimer [77, 80, 81]. Two Nop5/fibrillarin heterodimers further dimerize to form a heterotetramer [77, 80, 84]. Therefore, Nop5 and fibrillarin may assemble onto the sRNP complex in a tetrameric form [77]. Because fibrillarin contains an S-adenosylmethionine (SAM) binding pocket and has a conserved SAM-dependent methyltransferase fold [74, 77, 80, 84, 91], it is suggested to be the catalytic enzyme for 2′-*O-*methylation. Mutations in *S. solfataricus *and *S. cerevisiae *fibrillarin result in a loss of sRNP 2′-*O-*methylation activity [26, 92].

 Structural studies of archaeal box C/D sRNP individual core proteins, protein-protein, and protein-RNA complexes have revealed important structural features that are essential for assembly and catalysis [16, 68, 70, 74, 77, 84, 86, 89]. A cocrystal structure of L7Ae and a minimal box C/D sRNA confirmed the formation of a kink-turn motif and the importance of this motif in the binding of the L7Ae protein [70]. Crystal structures and biochemical studies of Nop5/fibrillarin complexes as well as a partial C/D sRNP complex assembled with L7Ae, Nop5, fibrillarin, and a half-mer box C/D sRNA revealed the importance of Nop5 protein as a protein bridge [77, 84, 86, 87]. The C-terminal domain (CTD) of the Nop5 protein contacts the composite surface formed by L7Ae and the sRNA while the N-terminal domain (NTD) of Nop5 interacts with fibrillarin [77, 84, 86, 87]. Furthermore, the coiled coil domain of Nop5 provides an interaction surface with another Nop5 protein, which serves as a mediator between the proteins assembled on two different box C/D motifs. Importantly, cocrystal structures of Nop5/fibrillarin complexes from different species as well as a partial C/D sRNP complex have suggested that there is a flexible hinge region in the Nop5 protein, around which the NTD moves [77, 84, 87]. This hinge region may also allow movement of the associated fibrillarin enzyme, allowing efficient catalysis upon substrate binding.

 Recently, the structure of a complete, catalytically active box C/D sRNP was successfully reconstructed for *M. jannaschii* using negative staining electron microscopy (EM) [68, 89]. Unexpectedly, this structure is a dimeric sRNP structure (di-sRNP) (Figure 5(a)), which is further supported by extensive biochemical evidence [68, 93]. In the di-sRNP model, rather than having two sets of the three core proteins associated with a single sRNA, a complete box C/D sRNP is composed of four sets of the core proteins associated with two sRNAs (Figure 5(a)). Intriguingly, the docking of crystal structures of the core proteins into the di-sRNP volume also suggested that the Nop5 dimers act as a bridge between the L7Ae and fibrillarin proteins assembled on the box C/D motif of one sRNA with those assembled on the box C′/D′ motif of a different sRNA. Thus, the sRNA does not run parallel to the Nop5 dimer (Figure 5(a)). While the relatively low resolution of the EM reconstruction does not provide atomic details, it does present a novel model of how the box C/D sRNP may carry out catalysis. 

 The di-sRNP model has been challenged by the crystal structure of a fully assembled sRNP from *S. solfataricus *that contains a substrate RNA [17]. In contrast to the di-sRNP structure, the crystal structure suggests that a complete sRNP complex is composed of two sets of the three core proteins assembled on a single sRNA (Figure 5(b)). This model, which is referred to as the mono-sRNP model, not only contrasts with the di-sRNP model in terms of oligomeric state of the complex but also suggests a different orientation of the proteins and sRNA in the complex. While the Nop5 dimer connects the box C/D (C′/D′) motifs from two sRNAs in the di-sRNP structure, the dimer connects the box C/D (C′/D′) motifs from the same sRNA in the mono-sRNP structure (Figure 5). The sRNA, therefore, runs parallel to the Nop5 dimer in the mono-sRNP model, which is distinct from the di-sRNP model.

 It was surprising that box C/D sRNPs from different archaeal species with very conserved protein components can adopt such diverse structures. One explanation may lie in the different sRNAs used. In the di-sRNP structure, a natural sRNA made of a single continuous strand containing both kink-turn and kink-loop motifs was used [68, 89]. In contrast, in the mono-sRNP structure, a nonnatural sRNA made of two strands of RNA, containing two kink-turn motifs and lacking an internal loop, was used [17]. This seemingly slight difference in the presence of the loop of the sRNA induces different sRNP structures as demonstrated by native gel experiments [16]. While the natural sRNA, with the internal loop, forms only the di-sRNP species, the use of a nonnatural sRNA, lacking an internal loop, leads to the formation of a mixed population of both mono- and di-sRNP species [16]. Protein components from four different archaeal species assemble both mono- and di-sRNPs, depending on the sRNA [16]. 

Since the di-sRNP structure contains a physiological sRNA, it will be important to determine whether it changes conformation upon binding of substrate RNA. While the crystal structure of the mono-sRNP complex provides information about substrate RNA interactions, it remains unclear how the di-sRNP structure changes conformation upon substrate binding. Because the orientation of the proteins and sRNA is fundamentally different between the mono- and the di-sRNPs, different conformational changes might therefore be expected upon substrate binding. Further structural studies on the box C/D di-sRNP complex assembled using a natural sRNA in the presence of the substrate RNA need to be performed to understand the conformational changes associated with RNA methylation catalysis.

## 11. Properties and Secondary Structures of Box H/ACA sRNA 

The box H/ACA sRNA is the sRNA component of the box H/ACA sRNP that catalyzes pseudouridylation. Most archaeal box H/ACA sRNAs consist of a ~65–75 nt single hairpin structure which is immediately followed by the consensus sequence ANANNA (box H) or ACA (box ACA) (Figure 4(b)) [37, 43, 59, 61, 64, 66]. However, box H/ACA sRNA containing two or three hairpins have also been reported in Archaea [64–66]. Similar to the archaeal box C/D sRNA, box H/ACA sRNA also contains a kink-turn or a kink-loop motif in the internal region of the RNA [64–66]. In the middle of the hairpin, there are two unpaired strands. These sequences can form two 4–8 nt duplexes with the substrate RNA where the site of modification remains unpaired and sandwiched between the two duplexes (Figure 4(b)) [27, 28, 30, 58–60, 94, 95]. This bipartite loop structure is known as the pseudouridylation pocket. The site of pseudouridylation is ~14–16 nt away from the box H or box ACA [27, 28, 30, 60].

## 12. Box H/ACA sRNPs

 Box H/ACA s(no)RNPs direct site-specific pseudouridylations in pre-rRNAs. The first significant advance in our understanding of archaeal box H/ACA sRNP function came from *in vitro *reconstitution of catalytically active box H/ACA sRNPs from recombinant archaeal proteins and *in vitro *transcribed sRNAs [27, 28]. These studies provided insight into the minimal set of proteins necessary for the assembly of a functional box H/ACA sRNP. 

 The minimal box H/ACA sRNP contains a set of four core proteins: L7Ae, Nop10, Gar1, and Cbf5 [27, 28, 76]. Biochemical studies have provided information on core protein interaction and assembly with box H/ACA guide sRNAs [27, 28, 76]. L7Ae, an important component of archaeal box C/D sRNPs, also recognizes the kink-turn or kink-loop motif of the box H/ACA sRNA [76, 96]. In contrast to the box C/D sRNP where Nop5 and fibrillarin are unable to bind to the sRNA prior to L7Ae, the box H/ACA sRNP core protein, Cbf5, is able to bind to the sRNA independently of L7Ae [27, 28]. Cbf5, based on its homology to the TruB family of pseudouridine synthases, is likely the catalytic moiety of the box H/ACA sRNP [94, 95, 97, 98]. Indeed, genetic depletion of the yeast Cbf5 homolog results in the loss of pseudouridylation of pre-rRNA [99]. Efficient binding of Cbf5 to the sRNA requires the box H or ACA motif, the pseudouridylation pocket, and a GAG sequence in the terminal loop [27, 28]. The binding of Cbf5 to the sRNA is necessary for the assembly of Nop10 and Gar1 with the sRNA [27, 28].

**Figure 6:**
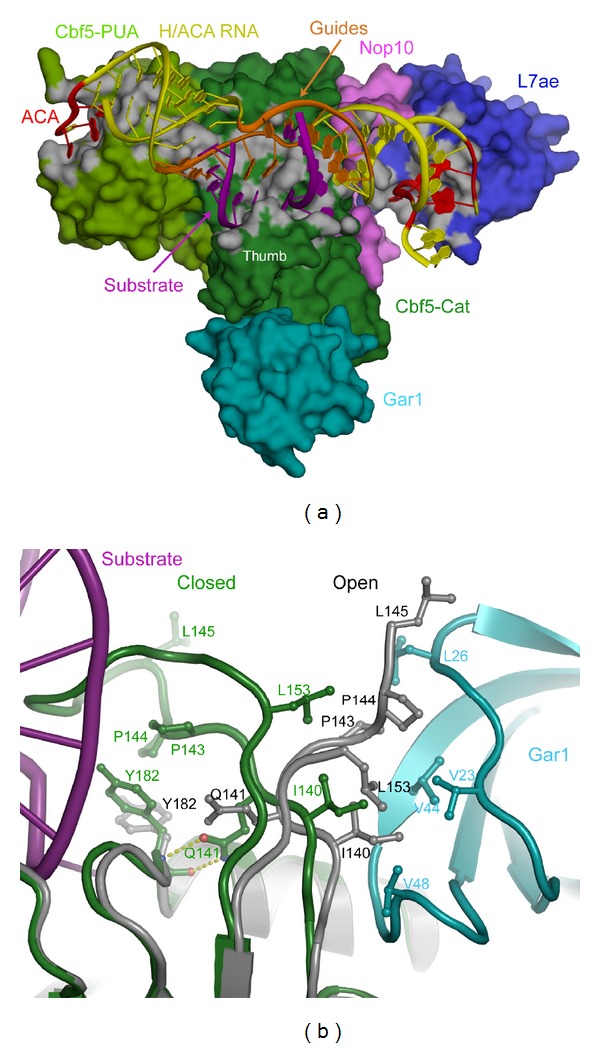
Structure of an archaeal box H/ACA sRNP (adapted from [52]). (a) The surface representation of the X-ray crystal structure of *P. furiosus* box H/ACA sRNP. The catalytic domain of Cbf5 (Cbf5-Cat) is in dark green, the PUA domain (Cbf5-PUA) is in light green, L7Ae is in blue, Nop10 is in magenta, Gar1 is in cyan, sRNA guide sequence is in orange, box ACA is in red, the remainder of the sRNA is in yellow, and the substrate RNA is in red. (b) Comparison of conformations of the thumb loop in the substrate-bound and the substrate-free state. The thumb loop conformation in the substrate-free state is in gray and that in the substrate-bound state is in green.

 In addition to biochemical studies, various structures of partial or complete core box H/ACA sRNPs have been determined [52, 100–105]. These structures provide atomic details for box H/ACA sRNP assembly and catalysis. As predicted, L7Ae binds to the kink-turn or kink-loop motif in the terminal loop near the upper stem [96]. Cbf5 can be divided into two domains, the catalytic domain and the PUA domain. The catalytic domain of Cbf5 interacts with the upper stem (P1) of the sRNA and the pseudouridylation pocket (Figure 6) [52, 101, 104, 105]. Nop10 wedges between L7Ae and Cbf5 and interacts with both proteins [52, 101, 104]. Therefore, Nop10 acts as an organizer that mediates the interaction between L7Ae and Cbf5. The PUA domain of Cbf5 interacts with the lower stem (P1) of the sRNA. In addition, it also sequence-specifically recognizes the box ACA motif [52, 101, 104, 105]. It has been suggested that the interactions of the PUA domain with the lower stem and the interaction of the catalytic domain with the upper stem positions the pseudouridylation pocket such that the target modification site is positioned correctly in the active site of Cbf5 [52].

 Furthermore, the structure of a substrate-bound box H/ACA sRNP shows that the helix formed between the 3′ guide sequence and the 5′ segment of the substrate interacts with the catalytic domain of Cbf5 [52]. An important structural element of the catalytic domain, known as the thumb loop, makes extensive contacts with the substrate RNA and has been proposed to be important to lock the substrate RNA into place (Figure 6) [52]. These interactions, together with the interactions between the PUA domain of Cbf5 and the lower stem of the sRNA, allow the precise positioning of the target modification site in the active site of Cbf5, which may explain why the spacing between the target modification site and the box H or ACA motif is usually ~15 nt [52].

 While the other core proteins interact with the guide sRNA and the substrate RNA, Gar1 does not make any contacts with either RNA (Figure 6) [52, 101, 104, 105]. Rather, Gar1 interacts only with Cbf5. This observation is consistent with the fact that the box H/ACA sRNP still has detectable activity even in the absence of Gar1 [27, 52]. Interestingly, Gar1 is only able to interact with the thumb loop of Cbf5 in the substrate-free state but not in the substrate-bound state (Figure 6) [52, 101]. The thumb loop, therefore, switches between a closed state, which makes contact with the substrate, and an open state, which makes contacts with Gar1. This switching between the two states has led to the hypothesis that while Gar1 may not have a direct role in the chemistry of the catalytic reaction, it may have a role in facilitating the release of the product [52].

## 13. Other Functions of Box C/D and Box H/ACA sRNPs

While the function of archaeal box C/D and box H/ACA sRNPs for RNP-guided modification is relatively well understood, these sRNPs may also perform other functions. Some eukaryotic box C/D and box H/ACA snoRNPs perform functions other than RNA modification [106, 107]. One example is the eukaryotic U3 box C/D snoRNP. Using the guide snoRNA base-pairing capacity, U3 snoRNP helps cleave the 35S rRNA and may act as a chaperone in RNA folding [107–113]. Furthermore, there are other eukaryotic box C/D and box H/ACA snoRNPs that have evolved pre-rRNA processing functions [95, 106, 107]. 

While it is apparent that U3-like sRNAs are absent in Archaea [114], the ability of archaeal box C/D and box H/ACA sRNPs to perform functions other than nucleotide modifications remains a possibility. This may explain why the targets of nucleotide modification of some of the candidate box C/D sRNAs and box H/ACA sRNAs cannot be identified [42, 43, 61–66]. Therefore, some archaeal sRNPs may function in pre-rRNA cleaving or folding [69, 107, 115]. The hypothesis that sRNPs act as chaperones is consistent with the box C/D sRNP di-sRNP model. A dimeric sRNP can potentially allow base-pairing with four segments of pre-rRNAs using the four guide sequences, trapping nearby pre-rRNA sequences and avoiding the formation of premature RNA tertiary structures [16, 69]. The nonmodification functions of archaeal sRNPs are speculative. It will be interesting to see if these functions can be demonstrated for the archaeal sRNPs.

## 14. Functions of Nucleotide Modification

 The locations of the pseudouridylations and 2′-*O*-methylations have been mapped onto the ribosomes of certain Eukaryotes, Bacteria, and Archaea [35] and are located in functionally important regions of the ribosome. Therefore, chemical modification has been proposed to have important functions in protein translation. While the removal of individual modifications does not have significant effects on the growth of an organism, studies in *S. cerevisiae* have shown that global removal of all modifications led to severe growth defects [35, 92, 116, 117]. Moreover, mutations that led to the removal of certain targeted nucleotide modifications also resulted in severe growth and protein synthesis defects [116, 118, 119]. Furthermore, depleting or mistargeting certain modifications in the *S. cerevisiae *rRNA, particularly in regions that are predicted to be important for ribosomal activity and/or ribosome biogenesis, also resulted in defects in ribosome synthesis and in mRNA translation [116, 118–120]. These studies have only been performed in eukaryotes. However, since nucleotide modifications are also located in important regions of archaeal ribosomes, they are likely to have important functions for archaeal ribosomes as well.

 Accumulating evidence suggests that nucleotide modification is important in archaeal ribosome function. In *S. solfataricus* there is an increase in the number of nucleotide modifications when the growth temperature is increased, indicating nucleotide modification may have a stabilizing effect on the structure of the ribosome [36]. This is especially important for many Archaea that live in extreme conditions. Indeed, NMR studies on a small segment of the 23S rRNA that contains a 2′-*O-*methylated nucleotide suggested that the modification enforces a certain local conformation of nearby nucleotides, thereby reducing the conformational dynamics of the nearby structures in a varying range of pH [121]. Taken together, these studies suggest that rRNA modifications influence the stability of ribosomes. However, since current functional studies on rRNA modification in Archaea are very limited, the precise function of most modifications is still unknown.

## 15. Conclusion

A more thorough understanding of how Archaea regulate the process of modification of the 23S, 16S, and 5S rRNAs will come from further studies of the sRNPs that catalyze modifications. In these roles, further studies on the box C/D and box H/ACA sRNPs will yield a more complete elucidation of their structure, enzymatic activities, and conformational changes. There is still much to learn in terms of the chemical modification of nucleotides on the rRNA. Because the Archaea represent an untapped resource of knowledge, further research will yield many interesting and surprising results from this independent domain that is neither Bacteria nor Eukarya.

## Figures and Tables

**Table 1:** Ribosomal RNA (rRNA) and rRNA processing across the three domains.

	Archaea	Eukarya	Bacteria
rRNAs	23S, 5S (LSU); 16S (SSU)	28S, 5.8S, 5S (LSU); 18S (SSU)	23S, 5S (LSU); 16S (SSU)
Ribosome	70S	80S	70S
Large subunit (LSU)	50S	60S	50S
Small subunit (SSU)	30S	40S	30S
Presence of introns in rDNA genes?	In some species [20–24]	In some species	Rare* [25]
Main rRNA modifying machines	sRNPs [26–28]	snoRNPs [29–33]	Site-specific protein enzymes [34, 35]
Number of rRNA Nms and Ψs	*S. solfataricus*: ~76 [36]	Yeast: 99 Human: 202 [34, 37–40]	*E. coli*: 14 [34, 37, 38, 40, 41]
Number of C/D and H/ACA s(no)RNAs	*S. solfataricus*: ~9–17 [42, 43]	Yeast: 75 Human: 207 [40]	0 [34, 35]

*Uncultured giant sulfur bacteria, such as *Thiomargarita namibiensis*, have been shown to contain self-splicing introns, varying in number and size, in the 16S rRNA gene. To date, this has been the only report of bacterial rDNA genes containing introns.
